# Supplementary material for: A Study on an Organic Semiconductor-Based Indirect X-ray Detector with Cd-Free QDs for Sensitivity Improvement
Source: Sensors (Basel). 2020 Nov 17;20(22):6562. doi: 10.3390/s20226562 (PMC7698411; doi:10.3390/s20226562)
Supplement: Supplementary file 1 [file sensors-20-06562-s001.pdf]

sensors

SUPPLEMENTARY INFORMATION

A Study on an Organic Semiconductor-Based Indirect X-Ray Detector with Cd-Free QDs for Sensitivity Improvement

Jehoon Lee, Hailiang Liu and Jungwon Kang *

Department of Electronic and Electrical Engineering, Dankook University, Gyeonggi-do 16890, Korea; usyj0512@gmail.com (J.L.); liuhailiang107@gmail.com (H.L.)

* Correspondence: jkang@dankook.ac.kr; Tel.: +82-31-8005-3624

Received: 21 October 2020; Accepted: 16 November 2020; Published: 17 November 2020

Section 1. Volume Percentage of InP QDs in the P3HT:PC$_{70}$BM with Embedded QDs and Calculation of the Average Distance Between QDs in the Film.

To calculate the distance between InP QDs [1], it was assumed that the InP QDs (3 mg) is uniformly dispersed in the active layer. The P3HT:PCBM solution was prepared by mixing a 1:1 ratio of P3HT and PCBM, and a total mass of 20 mg was prepared and dissolved in 700 μL of chlorobenzene (solution A). The InP QDs (10 mg) were dissolved in 1000 μL of chlorobenzene (solution B). The P3HT:PCBM:InP QDs solution was prepared by mixing solution A and B.

- <u>P3HT:PC$_{70}$BM volume calculation ($V_{P3HT:PCBM}$)</u>

  $d_{P3HT:PC70BM}$ = 1.3 g/cm^3 (= 1300 kg/m^3)
  The mass of the P3HT:PC$_{70}$BM in the chlorobenzene (solution A) is:
  $m_{P3HT:PC70BM}$ = 20 mg (20 x 10^{-6} kg)
  so, the volume of P3HT:PC$_{70}$BM in the chlorobenzene (solution A) is:
  $V_{P3HT:PC70BM}$ = 2 x 10^{-5} kg/1300 kg/ m^3 = 1.538 x 10^{-8} m^3

- <u>InP volume calculation (V_{InP})</u>

  d_{InP} = 4.81 g/cm^3 (= 4810 kg/m^3)
  The mass of the InP in the chlorobenzene (solution B) is:
  m_{InP} = 10 mg (10 x 10^{-6} kg)
  so, the volume of InP in the chlorobenzene (solution B) is:
  V_{InP} = 10^{-5} kg/4810 kg/ m^3 = 2.079 x 10^{-9} m^3

where, d is the density, m is the mass, and V is the volume. From the V_{InP} = 2.079 x 10^{-9} m^3, it was possible to calculate the InP volume contribution of the concentrations of 3 mg/ml in the solution and which was prepared by mixing 300 μL of solution A and 700 μL of solution B.

$V_{InP\ 3\ mg/mL}$ = (2.079 x 10^{-9} m^3) / (1000 μL/300 μL) = 6.237 x 10^{-10} m^3

Thus, the volume percentage is:

Vol% $_{\text{InP 3 mg/mL}}$ = $V_{\text{InP 3 mg/mL}}/V_{\text{P3HT:PC70BM}}$ = (6.237 x 10^{-10} m^3) / (1.538 x 10^{-8} m^3) x 100 = **4.05%**

- Calculation of InP-InP QDs distance

We assumed that the QDs are evenly distributed within the active layer. The volume of InP QDs was approximated to the volume of the sphere (4/3)πr^3.

V = I^3 = (Volume of one InP QDs) / (volume percentage)
I = [(Volume of InP QDs) / (volume percentage of InP in the active layer)]$^{1/3}$

- InP diamter 4 nm

$V_{\text{InP QDs}}$ = (4/3)πr^3 = 3.35 x 10^{-26} m^3
the distance is related to the volume percentage of InP in the active layer:
for 3 mg/mL (the volume percentage of 4.05%)
$I_{\text{InP 4 nm}}$ = [(3.35 x 10^{-26} m^3) / 0.0405]$^{1/3}$ = 9.56 nm
the border to border distance is = 9.11 nm – 4 nm = **5.56 nm**

- InP diamter 8 nm

$V_{\text{InP QDs}}$ = (4/3)πr^3 = 2.68 x 10^{-25} m^3
the distance is related to the volume percentage of InP in the active layer:
for 3 mg/mL (the volume percentage of 4.05%)
$I_{\text{InP 8 nm}}$ = [(2.68 x 10^{-25} m^3) / 0.0405]$^{1/3}$ = 19.11 nm
the border to border distance is = 18.2 nm – 8 nm = **11.11 nm**

- InP diamter 12 nm

$V_{\text{InP QDs}}$ = (4/3)πr^3 = 9.04 x 10^{-25} m^3
the distance is related to the volume percentage of InP in the active layer:
for 3 mg/mL (the volume percentage of 4.05%)
$I_{\text{InP 12 nm}}$ = [(9.04 x 10^{-25} m^3) / 0.0405]$^{1/3}$ = 28.65 nm
the border to border distance is = 27.3 nm – 12 nm = **16.65 nm**

Table S1. Distances between InP QDs for different sizes at 3 mg amount (4.05 vol%).

InP QD diameter [nm]	Between particles distrance [nm]
4	5.56
8	11.11
12	16.65

Section 2. Calculation Method of the Defect Density

Figure S1 is the J-V curve under dark room conditions to calculates the trap density, and the defect density equation is as follows:

$$N_{defects}\ (defect\ density) = \frac{2\varepsilon_r \cdot \varepsilon_0 \cdot V_{TFL}}{q \cdot L^2} \quad (1)$$

where $N_{defects}$ is the defect density, ε_r is the dielectric constant of the P3HT:PC_{70}BM:InP QDs, ε_0 is the permitivity of free space (8.854 x 10^{-4} F/m), q is the elementary charge (1.602 x 10^{-19} C), and L is the thickness of the P3HT:PC_{70}BM:InP QDs film. Also, V_{TFL} is defined as trap filled limit voltage, which represents the voltage at the point where the Trap-limited SCLC and Trap-filled limit region overlap in the log scale J-V curve [2].

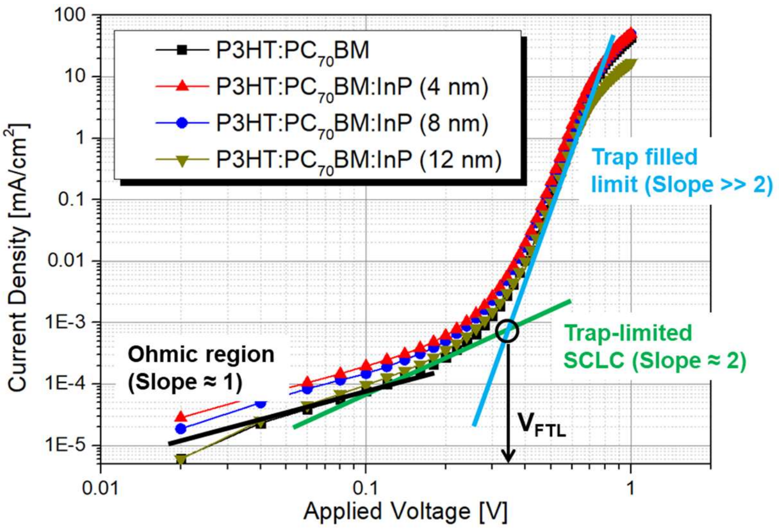

Figure S1. The space-charge limited current (SCLC) behavior of the detector with P3HT:PC_{70}BM and P3HT:PC_{70}BM:InP QDs (4-, 8-, and 12-nm).

Therefore, due to the addition of the same amount of InP QDs, the defect density is affected by V_{FTL}, and the calculated values are listed in Table S2.

Table S2. The defect density due to addition of different sized InP QDs to the P3HT:PCBM active layer.

Device	Defect density [cm^{-3}]
P3HT:PC_{70}BM	6.23 x 10^{15}
P3HT:PC_{70}BM:InP QDs (4 nm-diameter)	6.07 x 10^{15}
P3HT:PC_{70}BM:InP QDs (8 nm-diameter)	6.18 x 10^{15}
P3HT:PC_{70}BM:InP QDs (12 nm-diameter)	6.38 x 10^{15}

Reference

1. Masi, S.; Echeverria-Arrondo, C.; Salim, K. M. M.; Ngo, T. T.; Mendez, P. F.; Lopez-Fraguas, E.; Macias-Pinilla, D. F.; Planelles, J.; Climente, J. I.; Mora-Sero, I., Chemi-Structural Stabilization of Formamidinium Lead Iodide Perovskite by Using Embedded Quantum Dots, ACS Energy Lett. 2020, 5, 418-427.

2. Jahandar, M.; Khan, N.; Lee, H. K.; Lee, S. K.; Shin, W. S.; Lee, J. C.; Song, C. E.; Moon, S. J., High-Performance CH_3NH_3PbI-Inverted Plannar Perovskite Solar Cells with Fill Factor Over 83% via Excess Organic/Inorganic Halide, *ACS Appl. Mater. Interfaces* **2017**, *9*, 35871-35879.
